# Drivers of tropical rainforest composition and alpha diversity patterns over a 2,520 m altitudinal gradient

**DOI:** 10.1002/ece3.5155

**Published:** 2019-04-16

**Authors:** Dario Veintimilla, Marie Ange Ngo Bieng, Diego Delgado, Sergio Vilchez‐Mendoza, Nelson Zamora, Bryan Finegan

**Affiliations:** ^1^ CATIE-Centro Agronómico Tropical de Investigación y Enseñanza Turrialba Costa Rica; ^2^ CIRAD, UR Forêts et Sociétés, CIRAD Campus International de Baillarguet Montpellier Cedex 5 France

**Keywords:** alpha diversity, Beta diversity, dispersal assembly, Hill numbers, niche assembly, rarefaction‐extrapolation, tropical mountain forest

## Abstract

**Aim:**

We sought to determine the relationship of forest composition and alpha diversity (the species diversity of a local assemblage) to altitude, soil, and spatial factors over a 440–2,950 m a.s.l gradient.

**Location:**

Altitudinal gradient on the Caribbean slope of the Talamanca Cordillera, Costa Rica.

**Taxon:**

Angiosperm and gymnosperm trees, palms, and tree ferns.

**Methods:**

We measured and identified all stems ≥10 cm dbh in 32 0.25‐ha undisturbed rain forest plots over the gradient. We determined compositional patterns using Non‐Metric Multidimensional Scaling (NMS) ordination, and used linear regressions to explore the relationship between four alpha diversity metrics and altitude. With variation partitioning (VARPART), we determined the compositional variation explained by altitude, soil, and spatial variables quantified using Principle Components of Neighbor matrices.

**Results:**

We identified 425 species. NMS axis 1 separated a lowland zone (440–1,120 m asl) from a transitional one dominated by holarctic *Oreomunnea mexicana* (1,400–1,600 m asl) and *Quercus*‐dominated forests at altitudes >2,100 m asl. The lowland zone was separated into two clusters of plots on NMS axis 2, the first in the 430–620 m asl range and the second at 1,000–1,120 masl. Regressions showed that all alpha diversity metrics were strongly negatively related to altitude (*R* ^2^ > 0.78). Overall, adjusted *R* ^2^ from VARPART was 0.43, with 0.30, 0.21, and 0.17 for altitude, soil, and space respectively. The respective adjusted *R* ^2^ of individual matrices, on controlling for the other two, was 0.06, 0.05 and 0.09 (*p* < 0.001).

**Main conclusions:**

There are two well‐defined forest compositional zones on this gradient—lowlands 430–1,120 m asl and montane forests >2,150 m asl—with a transitional zone at 1,400–1,600 m asl, where lowland tropical and montane holarctic species are found together. Montane forests are very distinct in their composition and low alpha diversity. Vegetation and soil respond to altitude, and therefore temperature, as an integrated system, a model that goes beyond niche assembly as shown by the significant effect of space in the VARPART.

## 1 INTRODUCTION

Tropical mountain forests (TMF) can be considered in a broad sense to be those forests located on mountains above 300 m asl (Korner, 2007). These TMF are vital ecosystems. Indeed, mountains cover a quarter of the Earth's land area. They are heterogeneous environments that support about a third of terrestrial plant diversity, and they supply half of humanity with drinking water (Korner, 2007). Tropical mountain forest ecosystems are gravely threatened; however, there is a critical need to understand their diversity and function in order to evaluate their potential response to global change drivers (Malhi et al., 2010). The potential effects of climate change and their interaction with other drivers are a particularly urgent area for study (Bush, Hanselman, & Hooghiemstra, 2011). In particular, the driving forces affecting the maintenance of high tropical mountain forest, beta and alpha diversity on altitudinal gradients are still not well understood (Silman, 2011).

Many studies have described the macro‐scale zonation of forests on tropical mountains in terms of forest structure and physiognomys (Gentry, 1995; Grubb, 1977; Holdridge, 1967; Whitmore, 1984). Zonation at this scale is related to altitude and climate, and temperature gradients are probably the main drivers of forest zonation in places where rainfall regimes do not vary significantly with altitude (Holdridge, 1967; Malhi et al., 2010). Climatic zones may or may not coincide with zonation of floristic composition. Thus, Kitayama (1992) identified four altitudinal vegetation zones on Mount Kinabalu, Borneo, made up by “mutually exclusive” groups of species. He interpreted this zonation as defined by “critical altitudes” linked to climatic thresholds. On the other hand, Lieberman, Lieberman, Peralta, and Hartshorn (1996), suggested that rain forest composition varies linearly with altitude over a 100 m to 2,600 m asl. altitudinal range in Costa Rica. The strong correlations between altitude, climate, and other environmental factors, especially soil, continue to represent a major challenge to complete understanding of the response of forest vegetation to altitude (Chain‐Guadarrama, Finegan, Vilchez, & Casanoves, 2012; Grubb, 1977; Malhi et al., 2010).

Theory provides complementary models for the explanation of compositional variation in tropical mountain rain forests. Compositional variation (beta diversity) over environmental gradients may be a consequence of differential adaptation of species to gradients of climate, soil, and other factors (niche assembly) together with the effects of geographical distance and dispersal limitation (dispersal assembly; Condit et al., 2002; Legendre et al., 2009; Potts, Ashton, Kaufman, & Plotkin, 2002). However, the lack of attention to tropical rain forest on altitudinal gradients in studies of the relative importance of community assembly models is notable (Chain‐Guadarrama et al., 2012; Tello et al., 2015). Most authors implicitly assume that the marked compositional change of forests on tropical mountains responds to a niche assembly model (Grubb, 1977; Homeier, Breckle, Gunter, Rollenbeck, & Leuschner, 2010; Lieberman et al., 1996; Macia, Ruokolainen, Tuomisto, Quisbert, & Cala, 2007; Tello et al., 2015). Niche assembly is certainly an attractive framework when marked changes in forest composition parallel steep gradients of climate, soil, and ecosystem process rates (Bruijnzeel & Veneklaas, 1998; Grubb, 1977; McGroddy & Silver, 2011; Tanner, Vitousek, & Cuevas, 1998; Wilcke et al., 2008). Besides the obvious atmospheric temperature gradients, the variation of soils with altitude is reasonably predictable. Soil organic matter and acidity tend to increase with altitude (Roman, Scatena, & Bruijnzeel, 2010). Tanner et al. (1998) speculate that phosphorous is the primary limiting element in lowland soils, while a high C/N ratio in montane soils may mean that N is a limiting factor, see also Reich and Oleksyn (2004) and Benner, Viitousek, and Ostertag (2010). One study nevertheless suggests that dispersal assembly may play a role in the explanation of beta diversity (Chain‐Guadarrama et al., 2012).

Few studies have attempted to separate the influence of climate from the influence of soil, and possible spatial effects (dispersal limitation) on tropical forest composition on altitudinal gradients. Generalization is made difficult by the different altitudinal ranges sampled and the use of a variety of protocols. Chain‐Guadarrama et al. (2012) sampled trees >30 cm dbh and palms >10 cm dbh in 0.25 ha plots on a 0–1,500 m asl gradient in Costa Rican wet forests, finding that spatial variables and soil (percent of clay and the cations Ca and Mg) had the strongest influences on forest composition. Conversely, sampling individuals >2.5 cm dbh in 0.1 ha plots, Arellano et al. (2016) found that Worldclim climate variables were always stronger predictors of variation of woody species composition than soil over a 4,000 m asl Andean altitudinal gradient.

Altitudinal changes of plant species alpha diversity in rain forest on tropical mountains are as marked as the changes of forest composition. Following Gotelli and Ellison (2013), we define alpha diversity as the species diversity of a local assemblage, in our case the trees, palms, and tree ferns in a sample plot or group of sample plots). At broad scales, the density of woody species (the number of species in sample plots (Gotelli & Colwell, 2001)) in tropical forests tends to increase with rainfall and soil fertility and to decrease with increasing seasonality, latitude, and altitude (Gentry, 1988a; Givnish, 1999; Silman, 2011; Ter Steege et al., 2003). Land area may be a major driver of patterns of organismic diversity on mountains (Bhatta, Grytnes, & Vetaas, 2018; Rosenzweig, 1995). While some studies suggest that density of woody species decreases linearly with altitude, others show a hump‐shaped relationship, with higher species density in foothill or mid‐altitude forests than in lowlands (Gentry, 1995; Girardin et al., 2014; Lieberman et al., 1996; Silman, 2011). The geometric constraints underlying the mid‐domain effect (Colwell & Lees, 2000) may help generate hump‐shaped relationships. Along the gradients where water is not a limiting factor, it is possible that declines of species density with altitude are correlated with declines in energy availability, either directly or indirectly through trophic cascades (Currie & Paquin, 1987; Hawkins et al., 2003; Huston, 2014; Rahbek, 1995). Theory also suggests that vegetation productivity may be related to species richness (Huston, 2014; Kessler & Kluge, 2008). Tropical forest productivity varies with altitude at a coarse scale, between lowland (<1,000 m asl) and upland (>1,000 m) forests (Cleveland et al., 2011), but relationships between this pattern and alpha diversity have not been explored. Finally, species density is undoubtedly influenced by disturbance regimes (Huston, 2014) but we are not aware of any study that attempts to characterize the variations of disturbance regimes across tropical rain forests on altitudinal gradients.

We conclude that understanding of control over patterns of forest composition and alpha diversity in tropical rain forests on altitudinal gradients is still limited by lack of data and direct hypothesis tests (Rahbek, 1995; Sanders & Rahbek, 2012; Silman, 2011). Therefore, the characterization of TMF beta‐ and alpha diversity and their relationship to environmental and spatial factors remains a key research area to which both real‐time studies and paleo‐ecological approaches must contribute (Bush et al., 2011).

In this study, we contribute to filling some of these knowledge gaps through an analysis of rain forest composition and alpha diversity and their relationship to environmental factors over a 440–2,950 m.a.s.l. altitudinal gradient on the Caribbean slope of Costa Rica's Talamanca Cordillera. We assume that water is not a limiting factor on this gradient. We first tested the hypothesis that because of the very marked environmental gradients in the study area, altitude (as a surrogate of mean annual temperature MAT) and soil characteristics are the main predictors of variation of forest composition, in comparison with spatial variables. We quantify space using the Principle Coordinates of Neighbor Matrices (PCNM) approach (Borcard & Legendre, 2002). Then, we determined whether alpha diversity shows a hump‐shaped relationship to altitude or whether it declines linearly as altitude increases. We sampled trees, palms, and tree ferns of diameter at breast height (dbh, 1.3 m) ≥10 cm in 32 plots of 50 m × 50 m (0.25 ha) distributed over the altitudinal gradient. Besides characterizing spatial relationships among plots using PCNMs, we measured soil conditions in each one.

## 2 STUDY AREA

**Figure 1:**
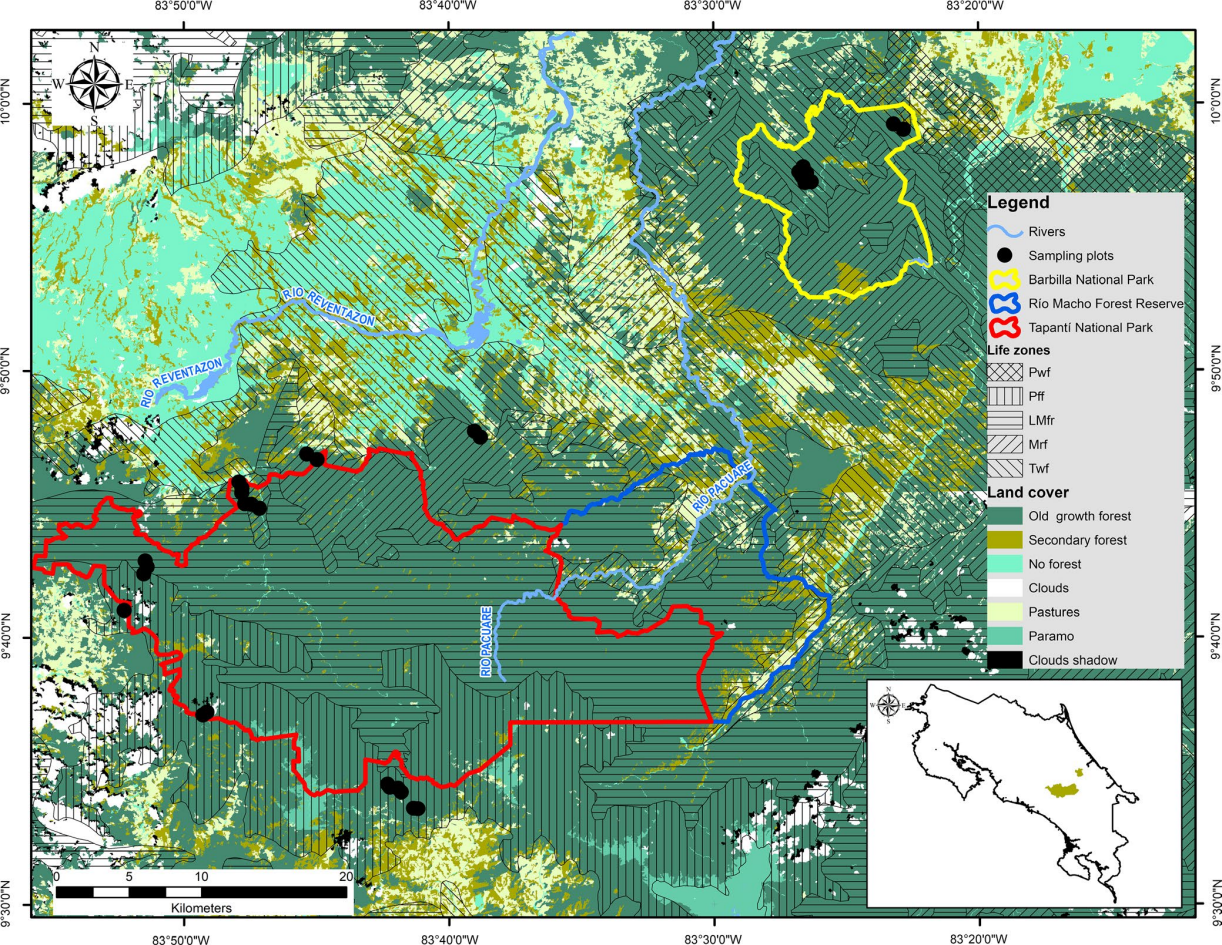
Map of the study area showing the location study 32 plots in the Caribbean slope of the Talamanca mountain range, Costa Rica

The research was carried out on the Caribbean slope of the Talamanca mountain range, Costa Rica (Figure 1, plot geographical coordinates and altitudes are in Supporting Information Appendix S1). This cordillera was formed during the Cretaceous from sedimentary, volcanic, and Miocenic plutonic rocks (Drummond et al., 1995). It is characterized by elongated crests and is deeply dissected by V‐shaped ravines (Berner, 1992; Blaser & Camacho, 1991). We sampled an altitudinal gradient ranging from 430 to 2,950 m asl. Worldclim data indicate that mean annual temperatures range from 24.4°C at 400 m asl to 10°C at 2,950 m asl on this gradient (Supporting Information Appendix S2). Annual rainfall is not correlated with altitude and from WorldClim data, ranges from 2,000 mm at the high end of the gradient to about 4,000 mm around 1,200 m asl. The Holdridge life zones represented in the sampled areas of the gradient (ITCR, 2008) are tropical wet forest, premontane rain forest, lower montane rain forest, and montane rain forest. *Quercus*‐dominated montane rain forests of the Talamanca Cordillera (2,000–3,200 m asl) were described by Kappelle, Vanuffelen, and Cleef (1995).

In national‐scale maps, ITCR (2008) identifies tropical wet forest and premontane rain forest soils as predominantly Ultisols belonging to the suborder Humult, and Inceptisols belonging to the suborders Tropept, Aquept, and Andept. In montane forests, soils are volcanically influenced Andepts characterized predominantly as Dystrandepts and Placandepts (Blaser & Camacho, 1991).

## 3 METHODOLOGY

### 3.1 Field sampling

#### 3.1.1 Sample plots

We established 32 permanent and temporary sample plots in state and privately owned protected areas along the altitudinal gradient. Due to the extensive deforestation of some sectors of the gradient and the difficulty of access to the mature forests, we distributed plots over seven sites as follows (see also the map Figure 1). Ten plots were established between 440 and 620 m asl in Barbilla National Park; four plots at 1,000–1,120 m asl in two private protected areas; six plots at 1,400–1,660 m asl in Tapantí National Park; and finally, twelve plots at 2,150–2,950 m asl in three sites in Tapantí National Park and Rio Macho Forest Reserve. Plot installation criteria followed Sesnie, Finegan, Gessler, and Ramos (2009) and Chain‐Guadarrama et al. (2012). At each site, undisturbed mature forest sites were selected. Horizontal distance between plots was at least 300 m, avoiding atypical terrain conditions. Plots were placed >50 m from watercourses and we avoided areas with slopes >100% (Sesnie et al., 2009). All plots were geo‐referenced using a GPS (Garmin GPSMAP‐60csx), and altitude was measured with a calibrated altimeter in m asl (Supporting Information Appendix S1).

#### 3.1.2 Vegetation sampling

In each plot, we measured the stem diameter at breast height (dbh, 1.30 m) of all trees, palms, and ferns dbh ≥10 cm. Botanical identification was made directly in the field by experienced parataxonomists, Vicente Herra and Marvin Mena, and foliage samples were taken from those individuals that could not be identified in the field for identification by Zamora (Chain‐Guadarrama et al., 2012; Sesnie et al., 2009).

#### 3.1.3 Soil sampling and analysis

We sampled soils using a protocol developed for studies of vegetation–environment relationships in tropical forests and previously applied by Chain‐Guadarrama et al. (2012); Sesnie et al. (2009). In each plot, we collected a homogenized soil sample by mixing five subsamples taken from the four corners and center of each plot to a depth of 30 cm, eliminating the litter from the surface. Soil depth was measured with a 1.10 m‐long metal rod and recorded in four categories: deep, ≥90 cm; moderately deep, 50–90 cm; superficial 25–50 cm; very shallow <25 cm (Suárez de Castro, 1979). Slope (degrees inclination of each plot) was measured in five points with a clinometer and an average calculated for each plot. Physical and chemical analysis of soil was performed at the soil laboratory of the Agricultural Research Center (CIA) in the University of Costa Rica, San José. Samples were air‐dried. Soil texture (percent of sand, clay, and silt) was obtained by the Bouyoucos method (Beretta et al., 2014). Total acidity, Ca, and Mg extractions were obtained in 1 M potassium chloride (KCl). Extractable K, P, Zn, Cu, Mn, and Fe were obtained by modified Olsen extraction with a 0.5 N sodium bicarbonate (NaHCO_3_) solution at a pH 8.5. The percentages of C and N were determined by the autoanalyzer of C/N by dry combustion. Descriptive statistics of soil variables are presented as supplementary data (Supporting Information Appendix S3).

### 3.2 Analysis of data

#### 3.2.1 Variations of forest diversity and composition along the altitudinal gradient

In order to visualize the compositional relationships among plots, we performed a Non‐Metric Multidimensional Scaling (NMS) ordination analysis, using the Bray Curtis similarity index. This ordination was carried out using the abundance of the species present in each plots, for trees, palms, and tree ferns ≥10 cm dbh. The ordination was executed with 50 maximum number of iterations and a convergence tolerance of 0.00001. The convergence was obtained with 10 iterations. The analysis was performed with Qeco (Di Rienzo, Casanoves, Laura, Vilchez Mendoza, & Rienzo, 2010) in the R interface (R Development Core Team, 2017) with the libraries MASS and the isoMDS functions (Venables & Ripley, 2002).

We calculated four alpha diversity metrics. Species density was obtained directly for each sample plot. We calculated species richness and Shannon and Simpson diversity using Hill numbers (^0^D = species richness, ^1^D = Shannon entropy, ^2^D = Simpson diversity) estimating the effective number of species in each 0.25 ha plot (Hill, 1973; Jost, 2006). We used linear regression to explore the relationships between alpha diversity metrics and altitude. The calculated alpha diversity metrics with Hill numbers were performed with Qeco (Di Rienzo et al., 2010) in the R interface (R Development Core Team, 2017) with the libraries iNEXT (Hsieh, Ma, & Chao, 2016). Linear regression models were performed in InfoStat (J.A. Di Rienzo et al., 2017).

#### 3.2.2 Variation partitioning

In order to evaluate the percentage of variance in forest composition explained by soil, spatial variables, and altitude, we performed variation partitioning (VARPART; Borcard, Legendre, & Drapeau, 1992; Legendre, 2008; Peres‐Neto, Legendre, Dray, & Borcard, 2006). VARPART allows identification of “pure effects” of soil, spatial variables, and altitude, how much of the variation explained by environmental factors is spatially structured, and how much remains unexplained (Legendre, Borcard, & Peres‐Neto, 2005). We performed the Hellinger transformation on species abundances to decrease the weight of the most abundant species in the analysis (Legendre & Gallagher, 2001).

Spatial variables were obtained from the geographic coordinates of the plots transformed to a matrix of geographical distances between plots and using principal coordinate analysis of neighbor matrices (PCNM), following Borcard and Legendre (2002) and Dray, Legendre, and Peres‐Neto (2006). The analysis was performed in Qeco (Di Rienzo et al., 2010) in the R interface (R Development Core Team, 2017) with the libraries Vegan; PCNM function (Oksanen et al., 2013).

We used a forward selection procedure to select the soil and spatial (PCNM) variables most associated with the response matrix of species abundance per plot (Blanchet, Legendre, & Borcard, 2008). The hypothesis test was based on 10,000 permutations. The analysis was performed in Qeco (Di Rienzo et al., 2010) in the R interface (R Development Core Team, 2017) with the libraries Packfor (Dray, Legendre, & Blanchet, 2013).

We reported adjusted *R* ^2^ values (*R* ^2^_Adj_) indicating the proportion of variation explained by each of the predictor matrices (Peres‐Neto et al., 2006). The significance of variance fractions explained by each predictor matrix, as well as the individual and joint fractions, was tested with an analysis of variance by permutation test (*p* < 0.05, 10,000 permutations).

Analysis variations partition was performed in QEco (Di Rienzo et al., 2010) in the R interface (R Development Core Team, 2017) with the libraries vegan; varpart function (Oksanen et al., 2013).

## 4 RESULTS

In 32 sample plots of 0.25 ha (total area sampled 8.0 ha), we measured 4,261 individuals: trees, palms, and tree ferns >=10 cm dbh. We identified 425 species belonging to 92 plant families and 215 genera. From the 425 species identified, we identified 393 to species level, five to distinct morphospecies, and 27 to distinct taxa at the genus level.

4,088 individuals (96% of the total) were identified to species (including morphospecies) and 163 to genus. Most of individuals identified to genus were tree ferns (*Cyathea* spp.) in plots in the 1,400–1,600 m asl altitudinal range. Only 10 individuals were not identified because although alive, they lacked crowns. The list of all species of trees, palms, and tree ferns is provided as supplementary information (Supporting Information Appendix S4).

### 4.1 Variations of forest composition and alpha diversity over the gradient

**Figure 2:**
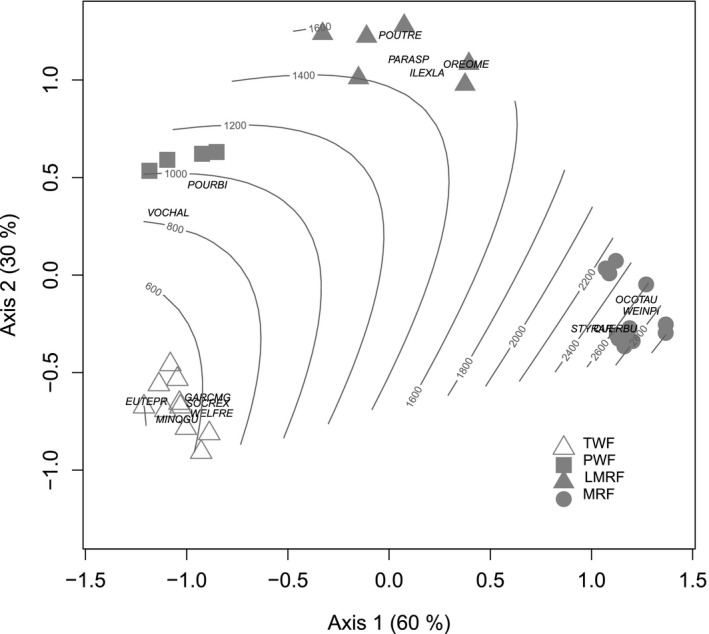
Non‐metric multidimensional scaling ordination diagram showing the location of vegetation plots along NMS axes one and two. Species better correlated to these axes of variation are shown (cross symbol): *Minquartia guianensis* (MINQGU), *Euterpe precatoria* (EUTEPR), *Socratea exorrhiza* (SOCREX), Welfia regia (WELFRE), *Garcinia magnifolia* (GARCMG), *Carapa guianensis* (CARAGU), *Hedyosmum scaberrimum* (HEDYSC), *Vochysia allenii* (VOCHAL), *Pourouma bicolor* (POURBI), characteristic of lowland forest up tol 1,100 m asl; *Pouteria reticulata* (POUTRE), *Oreomunnea mexicana* (OREOME), *Ilex lamprophylla* (ILEXLA), and *Parathesis crassiramea* (PARCR), characteristic of lower montane rain forest; and *Weinmannia pinnata* (WEINPI), *Ocotea austinii* (OCOTAU), *Styrax argenteus* (STYRAR), and *Quercus bumelioides* (QUERBU) characteristic of montane rain forest

A two‐dimensional solution was obtained from the NMS analysis of forest composition based on the abundances of 425 species recorded in each plots (Figure 2), with a final stress of 9.01. The ordinations suggest four floristically distinct forest types. Axis 1 separated plots in the 1,400–1,600 m asl and >2,100 m asl ranges from each other and from the plots in the 430–1,120 m asl range. Plots in this latter group were not separated from each other by axis 1, but were clearly separated by axis 2 (Figure 2). This is evidence that the main axis of compositional variation is made up by three zones. These zones are lowland plots below 1,120 m asl, high‐elevation plots >2,100 m asl, and a transitional zone at 1,400–1,600 m asl, suggesting that there is a distinct lowland flora below 1,100 m altitude on the gradient. Although each altitudinal zone has characteristic species associated with it (Figure 2), the axis 1 floristic spectrum is associated with species with broad altitudinal distributions shared between adjacent zones. For example, *Hedyosmum scaberrinum*, *Pourouma bicolor,* and *Vochysia allenii* were found in all the plots in the 430–1,120 m asl range, and *Drimys granadensis*, *Ocotea austinii,* and *Quercus bumelioides* across the 1,400–2,800 m asl range. In turn, the transitional plots at 1,400–1,600 m asl are made distinct by their unique dominant species—the tree fern *Alsophila firma*, and the canopy trees *Oreomunnea mexicana* and *Pouteria reticulata*, found nowhere else on the gradient. Finally, the two groups of lowland plots were differentiated from each other on NMS axis 2 by variation in the abundances of seven species found only in the 430–620 m asl range—the palms *Euterpe precatoria*, *Socratea exorrhiza*, *Welfia regia*, and *Iriartea deltoidea*, and the dicot tree species *Garcinia magnifolia*, *Minquartia guianensis*, and *Carapa guianensis*. These species had strong negative loadings on axis 2 of the ordination (Figure 2).

### 4.2 Species diversity

**Figure 3:**
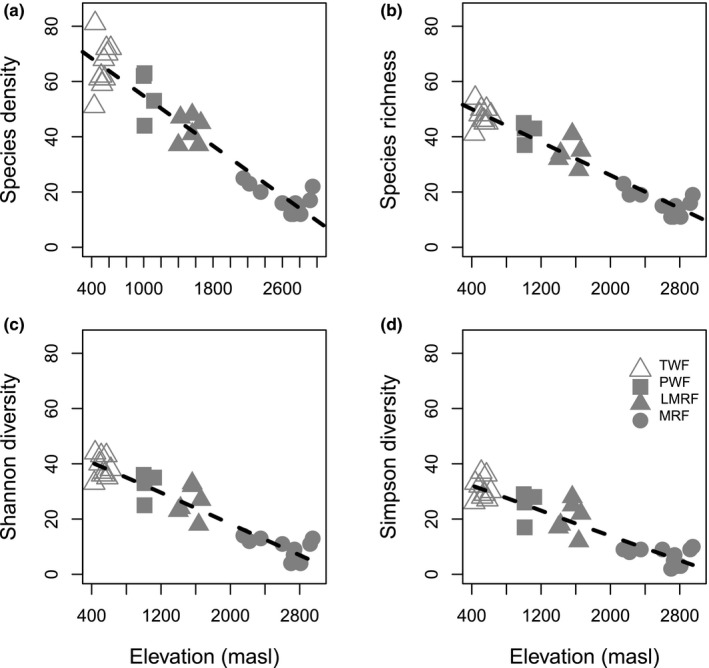
Relationships between altitude and (a) species density *R* ^2^ = 0.91, *p* < 0.0001; (b) species richness, Hill number ^0^ *D*: *R* ^2^ = 0.78, *p* < 0.0001; (c) Shannon entropy, ^1^ *D*:, *R* ^2^ = 0.87, *p* < 0.0001; (d) Simpson diversity, ^2^ *D*: *R* ^2^ = 0.82, *p* < 0.0001. Data for dicot trees, palms, and fern species ≥10 cm dbh in 32 0.25 ha sample plots. All effective numbers of species were standardized to 84 individuals

Species density and the effective numbers of species (for species richness and Shannon and Simpson diversity) showed linear declines with altitude, with *R* ^2^ = 0.91, 0.78, 0.87, 0.82, respectively (Figure 3a–d). The slope of the regression line was higher for species density than for the standardized diversity metrics (Hill numbers), suggesting an effect of stand density on species density in the lowland forests.

### 4.3 Variation partitioning of floristic composition by environmental variables

The selected variables were soils matrix, C/N, cation exchange capacity (CEC), acidity, pH, and % sand. The relationships of these soils variables to altitude are shown as supplementary Information (Supporting Information Appendix S3). For the spatial matrix, PCNMs 3, 4 and 30 were selected, representing both broad‐scale (PCNMs 3 and 4) and fine‐scale (PCNM 30) spatial relationships among plots.

**Figure 4:**
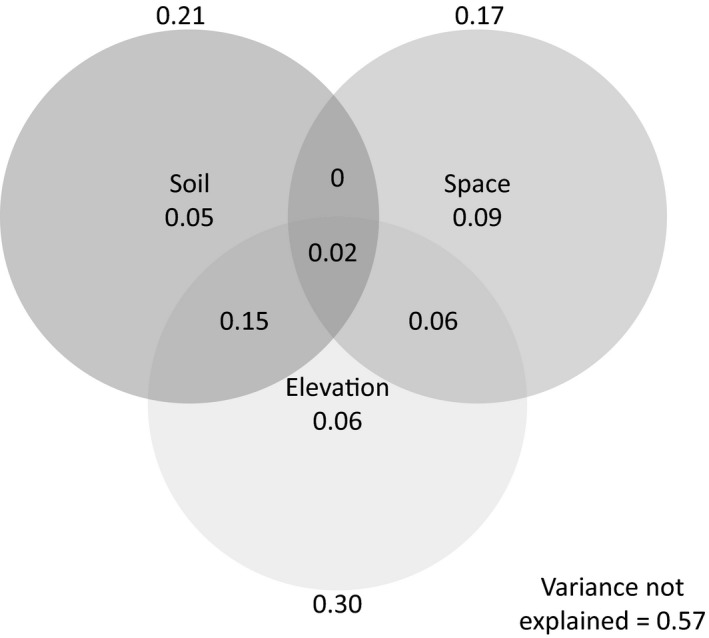
Variation partitioning results for predictor matrices of factors explaining variation in the floristic composition for trees, palms, and tree ferns >=10 cm dbh in 32 0.25‐ha plots on the study altitudinal gradient. Overall, adjusted *R* ^2^ was 0.43. Adjusted *R* ^2^ values, respectively, for soil, space, and altitude are shown for each predictor matrix for the overall effect (*R* ^2^_Adj_ = 0.21, 0.17, 0.30) and the “pure” effect of each matrix when controlling for the effects of all the others (*R* ^2^_Adj_ = 0.05, 0.09, 0.06)

The VARPART (Figure 4) showed that 43% of the variation of the abundance‐based composition of trees, palms, and ferns ≥10 cm dbh was explained by soil, space, and altitude. Overall, soil, space, and altitude explained 21%, 17% and 30% of the variation, respectively (Figure 4). However, “pure” effects of individual predictor matrices, though significant (*p* < 0.001), were all much smaller, with *R* ^2^_adj_ = 0.05, 0.09 and 0.06, respectively, for soil, space, and altitude. Shared effects between two variables when controlling for the third were 15% for soil and altitude and 6% for space and altitude; when controlling for altitude, soil, and space together were not significant.

## 5 DISCUSSION

For this 2,520 m altitudinal gradient in tropical rain forest, NMS ordination suggested a major axis of variation in forest composition among three groups of plots: lowland forests (sample plots at 440–1,120 m asl), plots at intermediate elevation where both lowland tropical and highland species were found (1,400–1,660 m asl), and montane forest (2,150–2,950 m asl). The compositional difference between the two groups of lowland plots is a secondary axis of variation: the many species shared between them (Supporting Information Appendix S4) lead to their not being separated on axis one of the ordination. A main factor underlying the separation of the two groups of lowland plots is variation of the abundance of four palm species, *Euterpe precatoria*, *Iriartea deltoidea*, *Socratea exorrhiza,* and *Welfia regia*. These palms are characteristic of Central American lowland rain forests on both Caribbean and Pacific slopes (Chain‐Guadarrama et al., 2012; Clark, Clark, Sandoval, & Castro, 1995; Sesnie et al., 2009). They were abundant in the 430–620 m asl range, but almost absent from the plots at 1,000–1,120 m asl. Palms do not respond to this altitudinal gradient in the same way as dicot trees, as Chain‐Guadarrama et al. (2012) also found, working on the Pacific slope of the Talamanca Cordillera. Some other characteristic lowland species of Costa Rica's Caribbean slope (Zamora, Artavia, Delgado, & Camacho, 1997) were absent from plots at 1,000–1,120 m asl (Supporting Information Appendix S4) while the dominants of these plots, *Pourouma bicolor* (Urticaceae), *Vochysia allenii* (Vochysiaceae), and *Calophyllum brasiliense* (Clusiaceae) were shared between the two lowland plot groups. The decline of palms between the two groups of plots is accompanied by a rise of tree ferns at 1,000–1,120 m asl (Supporting Information Appendix S4).

The composition of plots at 1,400–1,660 m asl is transitional between the lowlands and the highland plots. As on an Amazon‐Andes gradient studied by Gentry (1988b), species of Holarctic biogeographical affinity become dominant in these plots, in our case, *Oreomunnea mexicana* (Juglandaceae). However, some species were found across the whole altitudinal range 430–1,660 m asl, or the range 1,000–1,660 m asl. (Supporting Information Appendix S4). In this latter range, lowland species like *Alchornea latifolia*, *Billia rosea*, and *Cecropia insignis* are found together with *Oreomunnea mexicana* and species of Holarctic (*Quercus bumeliodes*) and austral‐Antarctic (*Drymis granadensis, Weinmannia* spp.) affinities (see Kappelle, Cleef, and Chaverri (1992) which become dominant in our plots at >2,150 m asl (Supporting Information Appendix S4).

The forest sampled in plots >2,150 m asl is compositionally very distinct from that of the rest of the gradient. It was characterized by constant species such as *Quercus bumelioides, Ocotea austinii, Styrax argenteus*, and *Weinmannia pinnata*, as shown by Kappelle et al. (1995). MRF also shows a very marked decline of species diversity in comparison with the other three life zones (see below).

Within the context of the compositional zonation and transitions shown by ordination, variation partitioning suggests overall control of composition by altitude, a surrogate of MAT, with an additional response mediated by soil. This interpretation would be consistent with a niche assembly model (Engelbrecht et al., 2007; Phillips et al., 2003; Potts et al., 2002; Pyke, Condit, Aguilar, & Lao, 2001; Ruokolainen, Tuomisto, Macia, Higgins, & Yli‐Halla, 2007; Sesnie et al., 2009). However, the combined effect of altitude and soil on controlling for space was the strongest of the three combined effects (*R* ^2^_adj_ = 0.15). Although Arellano et al. (2016) say that this combined effect is difficult to interpret, we suggest it indicates the operation of interactive control (Amundson & Jenny, 1997) through the strong relationship between MAT (measured by its surrogate altitude) and soil. Four of the soils variables selected for the variation partitioning—C/N, CEC, pH, and acidity—are correlated with the altitudinal gradient in regression analysis (Supporting Information Appendix S3). The small pure effects of both altitude and soil, together with their relatively strong combined effect, suggest that the vegetation–soil system responds in an integrated way to the state factor (Amundson & Jenny, 1997), temperature. Soil C/N has a negative linear relationship to community‐weighted mean specific leaf area in these plots (unpublished data), further supporting the integrated vegetation–soil response model. Finally, the pure effect of soil shown by VARPART likely reflects within‐life zone variability and future work on the importance of soil variability over altitudinal gradients may involve focus on the effects of this local variation.

Predominantly broad‐scale spatial effects reflect, overall, the spatialized nature of variation in altitude and soil (Arellano et al., 2016; Chain‐Guadarrama et al., 2012). One of the key contributions of variation partitioning, however, is to show the “pure” contributions of each matrix, on accounting for the effects of the other two (Legendre et al., 2009). In our study, the matrix of spatial variables had the highest pure effect, (*R* ^2^_adj_ = 0.09), suggesting either the operation of dispersal assembly or of unmeasured spatialized environmental variables (Chain‐Guadarrama et al., 2012; Condit et al., 2002; Sesnie et al., 2009).

Generalization about the relationship between alpha diversity and altitude is difficult because a range of sampling protocols has been used in other research, and altitudinal gradients with different starting points and lengths have been studied. Additionally, comparisons between Andean gradients and our study are potentially confounded by the mass effect, which could generate higher altitudinal limits of vegetation zones on the Andes than on Central American mountains (Grubb, 1977). Over our whole 2,520 m gradient, species density and effective numbers of species showed a very strong negative linear relationship to altitude. For species density, this result is consistent with previous neotropical studies, all of which measured only this alpha diversity metric (Gentry, 1988a,^1995^; Heaney & Proctor, 1990; Kappelle & Zamora, 1995; Lieberman et al., 1996; Silman, 2011). Our study also shows a negative linear diversity–altitude relationship using four alpha diversity metrics (Hill, 1973; Jost, 2006). Various mechanisms may explain the shapes of bivariate relationships between alpha diversity and altitude or temperature; alpha diversity seems likely to respond to a complex set of factors (see introduction). Tolerance of frost and chilling injury seems likely to be a key factor in the delimitation of the relatively small pool of species present at altitudes >2,150 m asl. The possession of such tolerance is suggested by the arctic and antarctic biogeographical affinities (Kappelle et al., 1992) of the dominant genera in these highland rain forests. The location and functional effect of the “frost line” (Holdridge, 1967) seems to have been neglected in research on tropical mountain forests.

We conclude the following. In terms of species composition, there are two well‐defined forest zones on this gradient—lowlands (430–1,120 m asl) and highlands (>2,150 m asl). The forests at 1,400–1,635 m asl are transitional as they share elements of both zones, as well as having a unique dominant species of holarctic affinity. This observed pattern of compositional variation is not aligned in a simple way with the four bioclimatic vegetation zones of the Holdridge system that are present on the gradient. This uncoupling of compositional zones from bioclimatic zones has two implications. First, that some tropical tree species are adapted to a broad range of bioclimatic environments on altitudinal gradients, which may confer resilience to climate change effects on such gradients. Second, that vegetation classifications based on bioclimatic zones and used in conservation planning and vegetation–climate modeling could be improved by recognition of actual floristic zones based on field sampling. The dominant life forms of lowland rain forests on both slopes of Costa Rica's Talamanca Cordillera—dicot trees and palms—do not respond to environmental gradients in the same way. The implications of these contrasting responses for understanding forest function also require further work. The highland forest is markedly distinct from the forest of the rest of the gradient in its composition and low alpha diversity, essentially representing a Holarctic intrusion into the geographically tropical Central American Isthmus. The role of tolerance of frost and chilling in sustaining this intrusion in the changing climate of the region are surprisingly little‐studied. In spite of the zonation of species composition, the relationship of forest alpha diversity to altitude, 430–2,950 m asl is linear. Variation partitioning suggests that species composition and soil characteristics respond to altitude, and therefore temperature, as an integrated system. Overall, control of species composition by temperature would be consistent with niche assembly. However, the integrated vegetation–soil response model goes beyond niche assembly. Species composition does not respond to fixed soil gradients, but interacts with and modifies the soil system. Responses to climate change should also be studied using a vegetation‐soil system approach. Further work is required to improve understanding of the potential influence of dispersal limitation on compositional variation in forests like these.

## CONFLICT OF INTEREST

The authors declare no conflict of interest exists.

## AUTHOR CONTRIBUTIONS

B.F., D.D, D.V., and S.V. conceived the research ideas. D.V., D.D., and N.Z. collected data. N.B.M.A. and B.F. led the writing. B.F., S.V., and N.B.M.A. advised on analysis. D.V., B.F., and S.V. performed statistical analyses of the data. All authors discussed the results and commented on the manuscript.

## Data Availability

The data supporting the results are archived in Dryad at: https://doi.org/10.5061/dryad.v9r72q7.
