# Supplementary material for: Drivers of tropical rainforest composition and alpha diversity patterns over a 2,520 m altitudinal gradient
Source: Ecol Evol. 2019 Apr 16;9(10):5720–30. doi: 10.1002/ece3.5155 (PMC6540655; doi:10.1002/ece3.5155)
Supplement: Supplementary file 1 [file ECE3-9-5720-s001.docx]

Appendix 1. Geographical coordinates (UTM05) and altitude for each of the 32 plots of 0.25 ha.

| **Sites** | **Plots** | **Coordinates** | | **Altitude m a.s.l** |
| --- | --- | --- | --- | --- |
|  |  | **W** | **N** |  |
| Parque Nacional Barbilla | PNB1 | 560610 | 1101413 | 490 |
| Parque Nacional Barbilla | PNB2 | 560715 | 1101056 | 550 |
| Parque Nacional Barbilla | PNB3 | 560912 | 1100767 | 620 |
| Parque Nacional Barbilla | PNB4 | 561140 | 1100308 | 570 |
| Parque Nacional Barbilla | PNB5 | 561013 | 1101390 | 440 |
| Parque Nacional Barbilla | PNB6 | 561221 | 1100598 | 580 |
| Parque Nacional Barbilla | PNB7 | 561248 | 1100976 | 520 |
| Parque Nacional Barbilla | PNB8 | 561569 | 1100363 | 540 |
| Parque Nacional Barbilla - Punta Lanza | PL1 | 567929 | 1103990 | 430 |
| Parque Nacional Barbilla - Punta Lanza | PL2 | 567241 | 1104363 | 510 |
| La Esperanza de Atirro | EA1 | 538740 | 1082737 | 1000 |
| La Esperanza de Atirro | EA2 | 538338 | 1083140 | 1010 |
| Reserva Biólogica el Copal | RBC1 | 527475 | 1081183 | 1010 |
| Reserva Biólogica el Copal | RBC2 | 526760 | 1081567 | 1120 |
| Parque Nacional Tapantí | PNT1 | 522072 | 1079639 | 1425 |
| Parque Nacional Tapantí | PNT2 | 522288 | 1079211 | 1560 |
| Parque Nacional Tapantí | PNT3 | 522284 | 1078881 | 1635 |
| Parque Nacional Tapantí | PNT5 | 523484 | 1077813 | 1400 |
| Parque Nacional Tapantí | PNT6 | 522935 | 1078099 | 1560 |
| Parque Nacional Tapantí | PNT7 | 522480 | 1078142 | 1660 |
| Parque Nacional Tapantí  - La Esperanza | PNTE1 | 515616 | 1074178 | 2150 |
| Parque Nacional Tapantí  - La Esperanza | PNTE2 | 515749 | 1073770 | 2220 |
| Parque Nacional Tapantí  - La Esperanza | PNTE3 | 515510 | 1073296 | 2350 |
| Parque Nacional Tapantí  - La Esperanza | PNTE4 | 514165 | 1070765 | 2600 |
| Reserva Forestal Río Macho- Villa Mills | VM1 | 533280 | 1058232 | 2700 |
| Reserva Forestal Río Macho- Villa Mills | VM2 | 533056 | 1058483 | 2810 |
| Reserva Forestal Río Macho- Villa Mills | VM3 | 532341 | 1058763 | 2740 |
| Reserva Forestal Río Macho- Villa Mills | VM4 | 532494 | 1058567 | 2780 |
| Reserva Forestal Río Macho- Villa Mills | VM5 | 534132 | 1057111 | 2750 |
| Reserva Forestal Río Macho- Villa Mills | VM6 | 534435 | 1057075 | 2730 |
| Reserva Forestal Río Macho- Tres de Junio | TJ1 | 519596 | 1063551 | 2950 |
| Reserva Forestal Río Macho- Tres de Junio | TJ2 | 519868 | 1063746 | 2920 |
