# Supplementary material for: Drivers of tropical rainforest composition and alpha diversity patterns over a 2,520 m altitudinal gradient
Source: Ecol Evol. 2019 Apr 16;9(10):5720–30. doi: 10.1002/ece3.5155 (PMC6540655; doi:10.1002/ece3.5155)
Supplement: Supplementary file 2 [file ECE3-9-5720-s002.docx]

Descriptive statistics of bioclimatic variables (from WorldClim) obtained for all 0.25 ha sampled plots along the altitudinal gradient in Costa Rica.

| **Bioclimatic variables** | **Code** | **Mean ±SD** | **Range** |
| --- | --- | --- | --- |
| Annual mean temperature (°C) | Tma | 17.89 ± 5.38 | 10-24.4 |
| Mean diurnal range (Mean of monthly (max temp – min temp) | Tmrd | 9.07 ± 0.9 | 7.9-10.4 |
| Isothermality (Tmrd/Tra) (* 100) | Ti | 7.99 ± 0.2 | 7.7-8.4 |
| Temperature seasonality (Standard deviation *100) | Te | 62.71 ± 7.97 | 52.2-76.2 |
| Max temperature of warmest month (°C) | Txcm | 23.68 ± 5.87 | 15.1-30.5 |
| Min temperature of coldest month (°C) | Tnfm | 12.43 ± 5.19 | 4.8-19.0 |
| Temperature annual range (Txcm - Tnfm) (°C) | Tra | 11.26 ± 0.88 | 10.2-12.6 |
| Mean temperature of wettest quarter (°C) | Tmht | 17.94 ± 5.2 | 10.3-24.5 |
| Mean temperature of driest quarter (°C) | Tmst | 17.92 ± 5.88 | 9.4-25.0 |
| Mean temperature of warmest quarter (°C) | Tmct | 18.61 ± 5.59 | 10.5-25.4 |
| Mean temperature of coldest quarter (°C) | Tmft | 17.04 ± 5.35 | 9.1-23.5 |
| Annual precipitation (mm) | Pa | 3213.16 ± 623.22 | 2338-4146 |
| Precipitation of wettest month (mm) | Phm | 406.19 ± 42.33 | 347-501 |
| Precipitation of driest month (mm) | Psm | 110.78 ± 64.27 | 29-210 |
| Precipitation seasonality (Coefficient of Variation) | Pe | 41.84 ± 15.19 | 22-63 |
| Precipitation of wettest quarter (mm) | Pht | 1136.03 ± 135.47 | 983-1462 |
| Precipitation of driest quarter (mm) | Pst | 377.28 ± 206.09 | 108-681 |
| Precipitation of warmest quarter (mm) | Pct | 717.44 ± 96.96 | 452-870 |
| Precipitation of coldest quarter (mm) | Pft | 644.97 ± 326.73 | 179-1055 |
| Evapotranspiration (mm) | Evapot | 1039.47 ± 355.69 | 535-1387 |
