# Supplementary figures and images for: Drivers of tropical rainforest composition and alpha diversity patterns over a 2,520 m altitudinal gradient
Source: Ecol Evol. 2019 Apr 16;9(10):5720–30. doi: 10.1002/ece3.5155 (PMC6540655; doi:10.1002/ece3.5155)

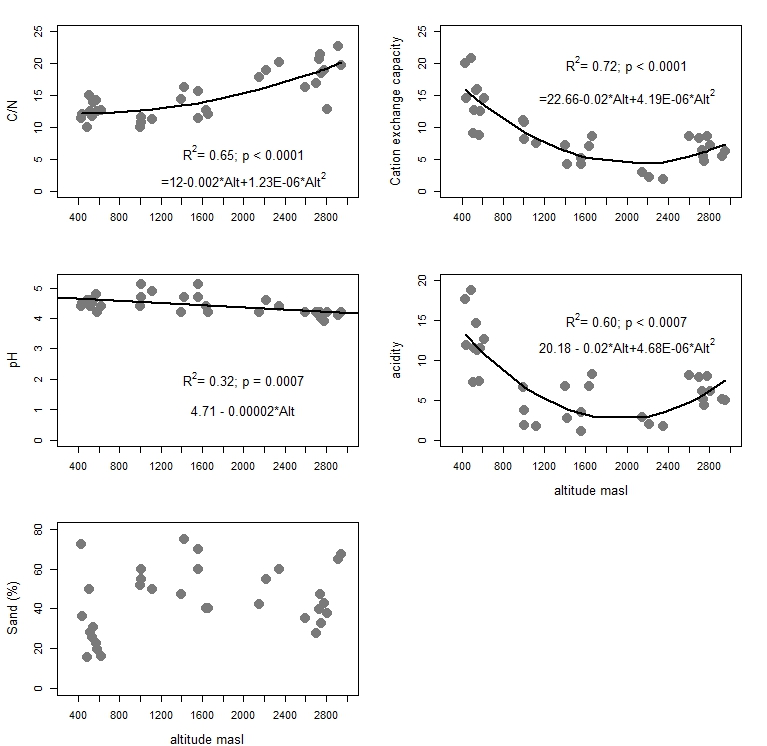

Supplement: Supplementary file 3 [file ECE3-9-5720-s003.tif]

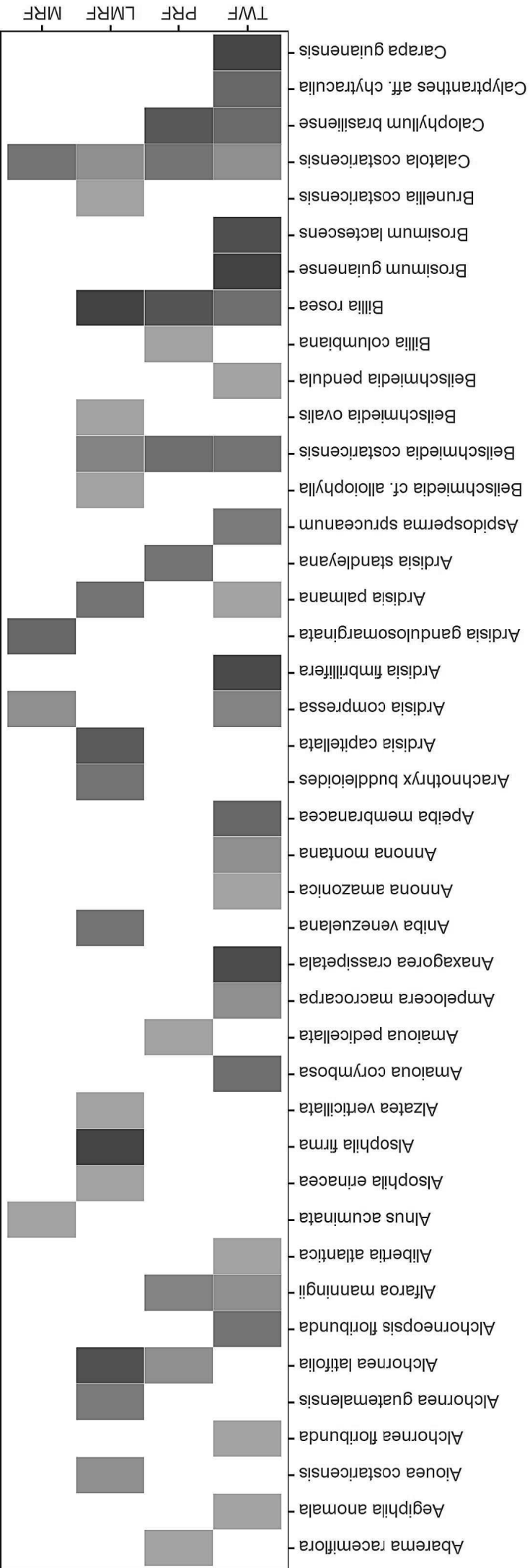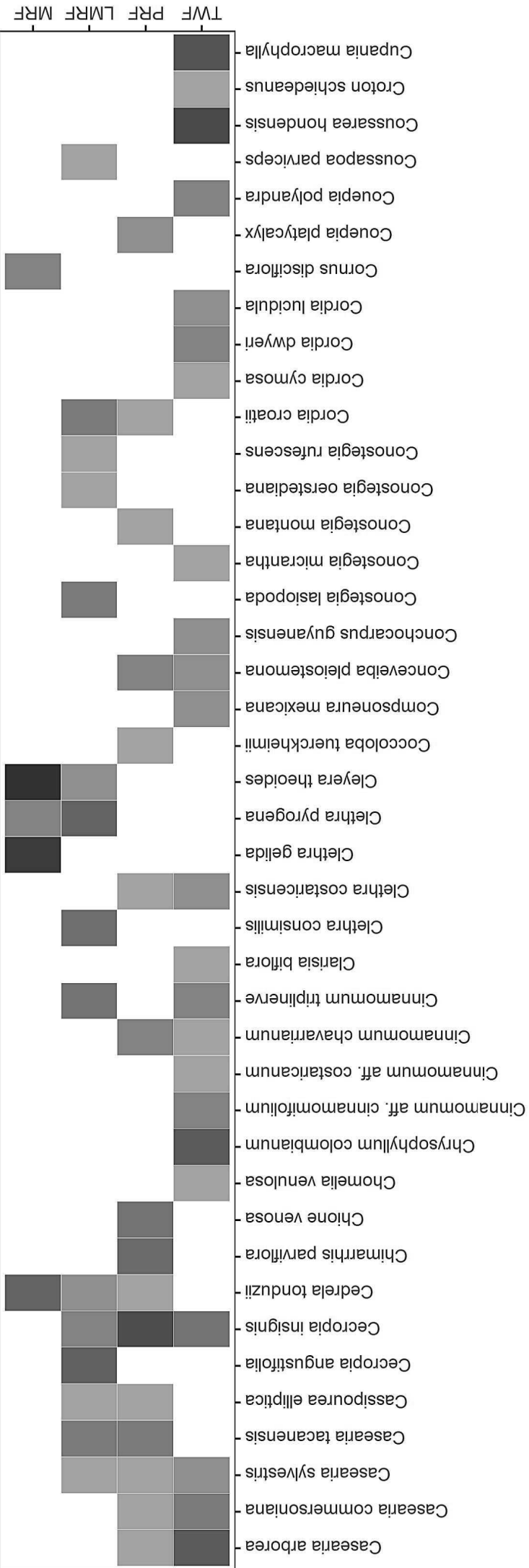

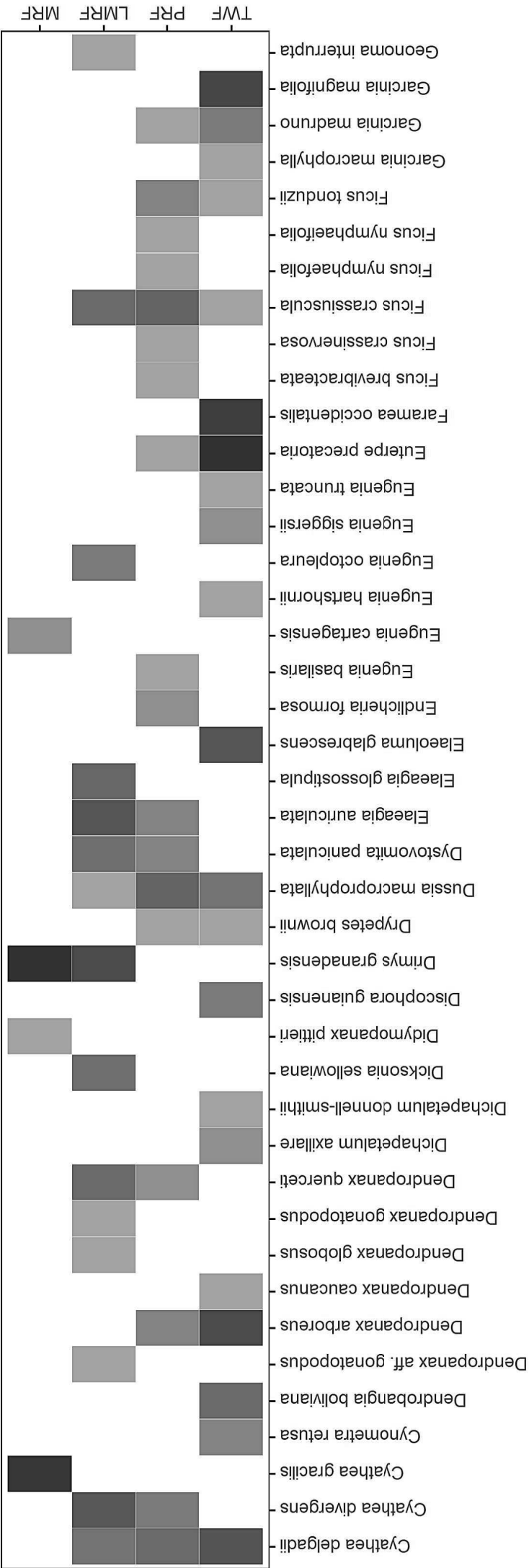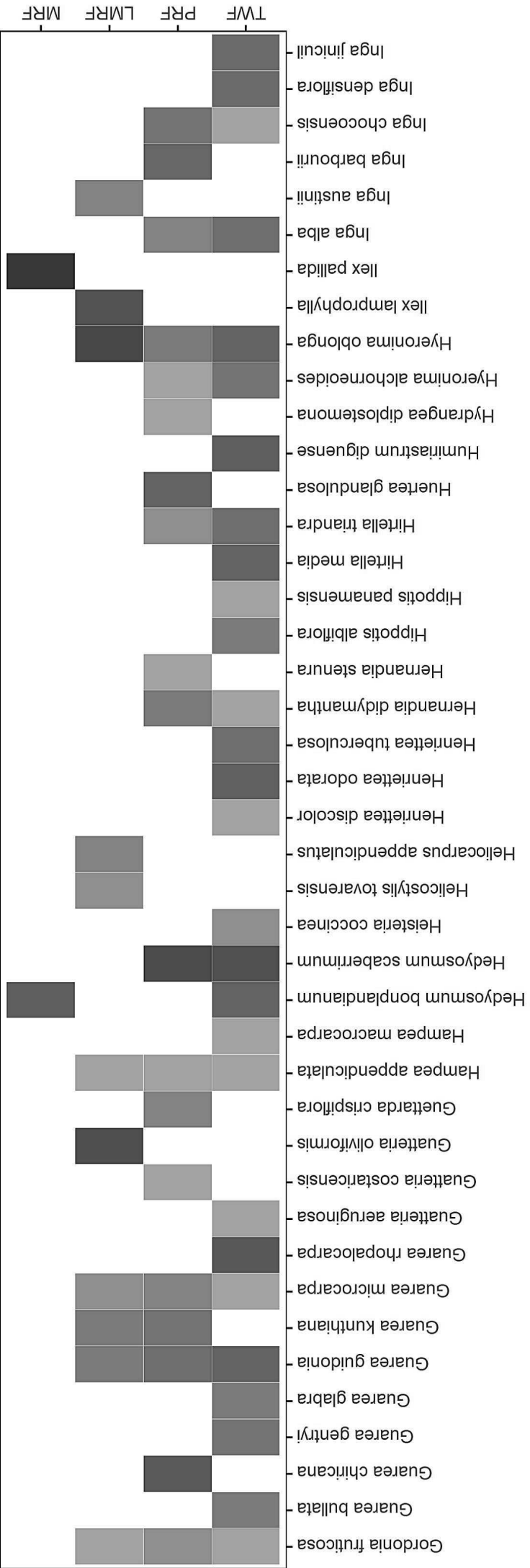

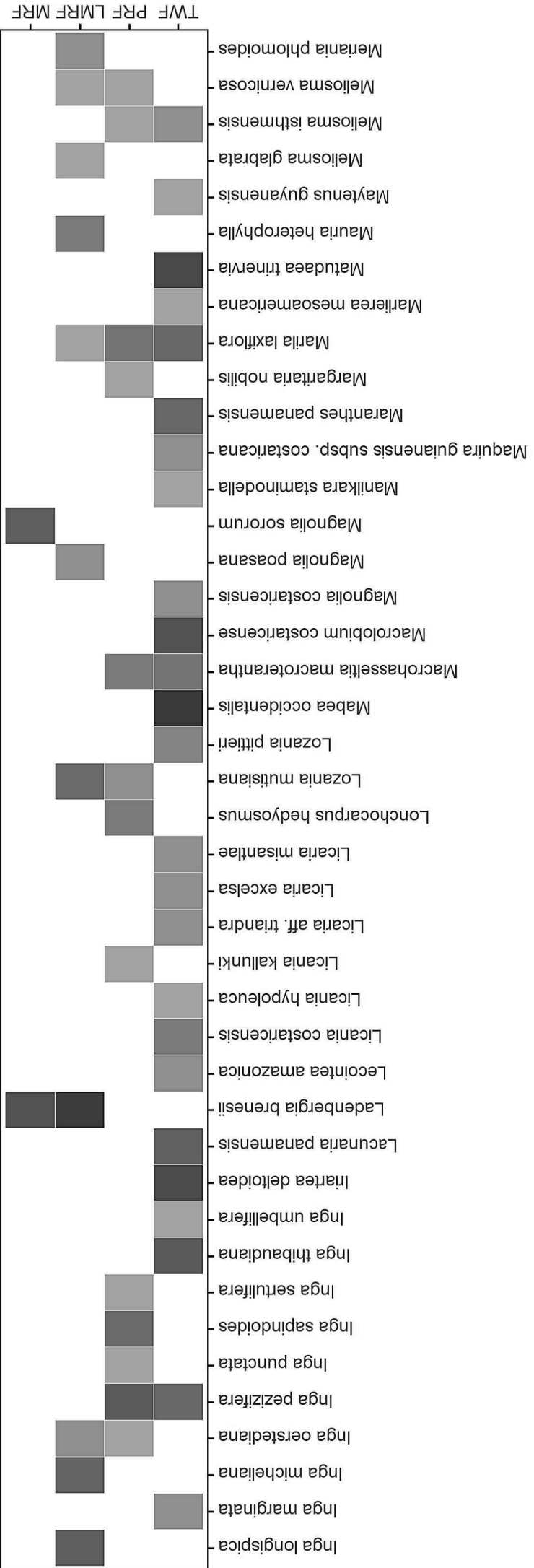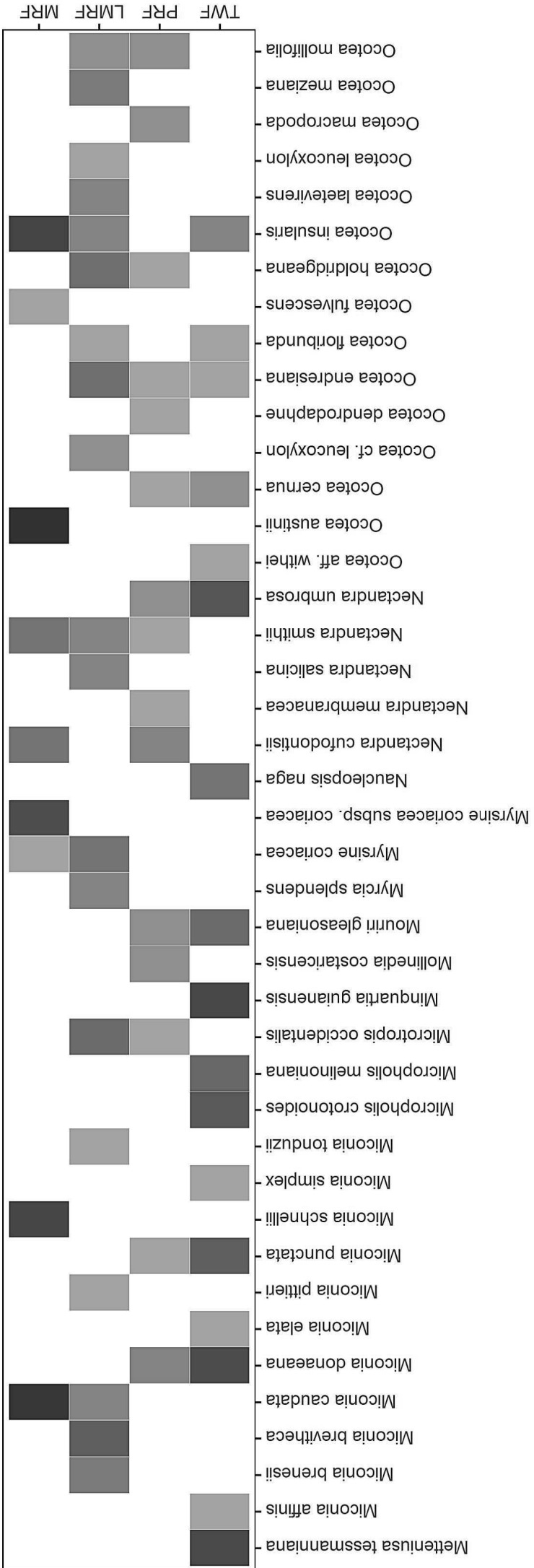

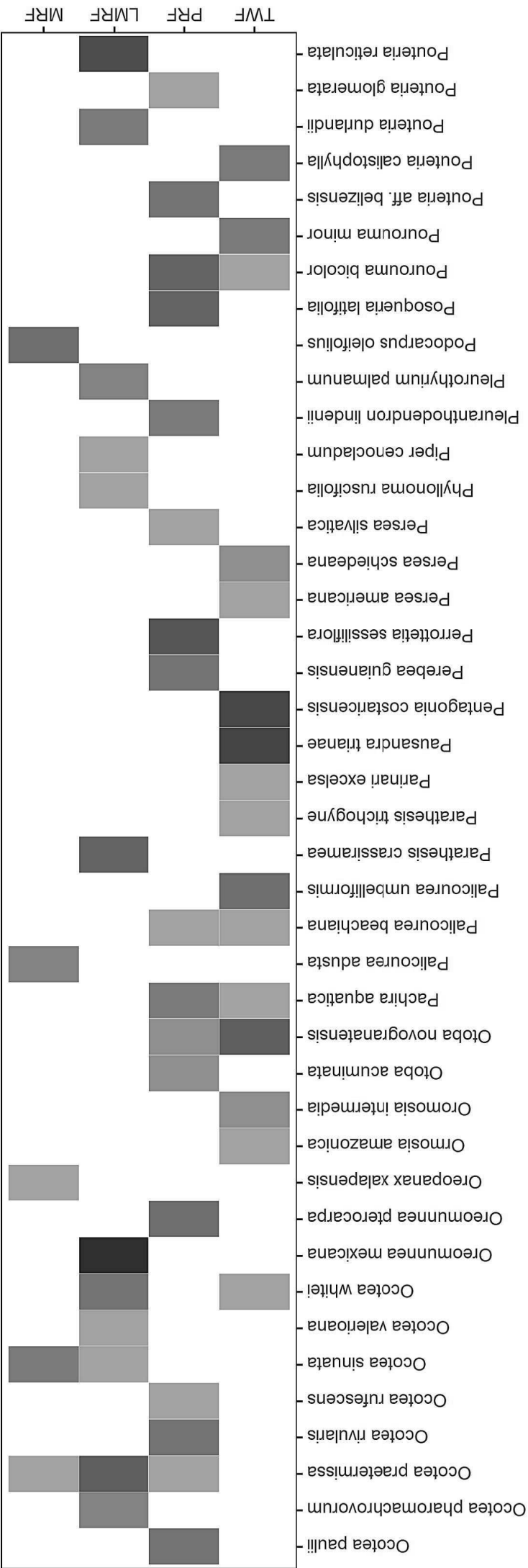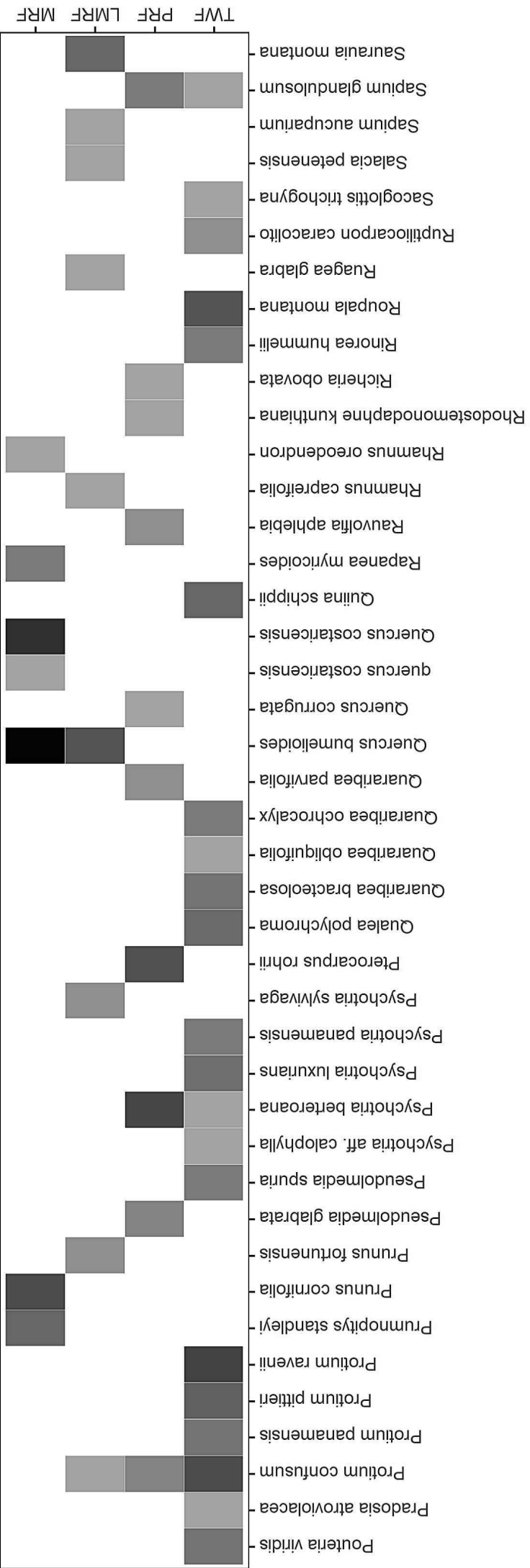

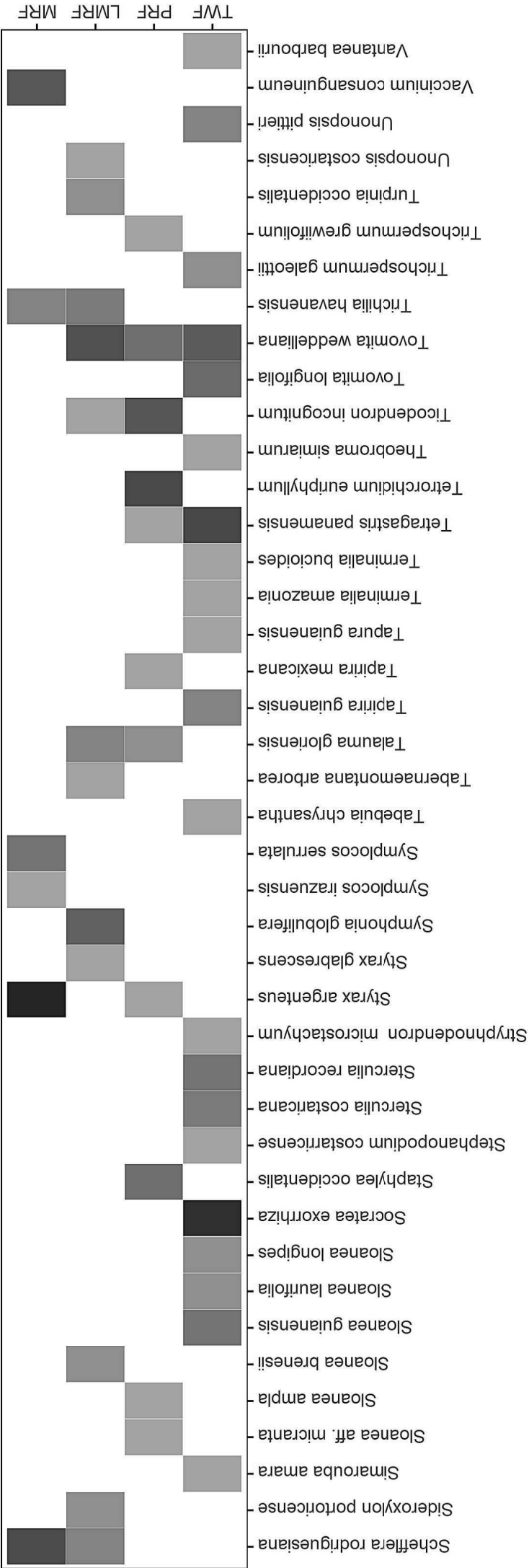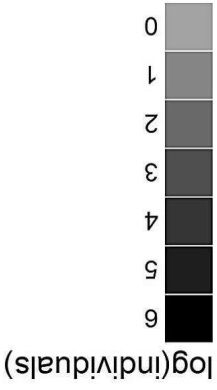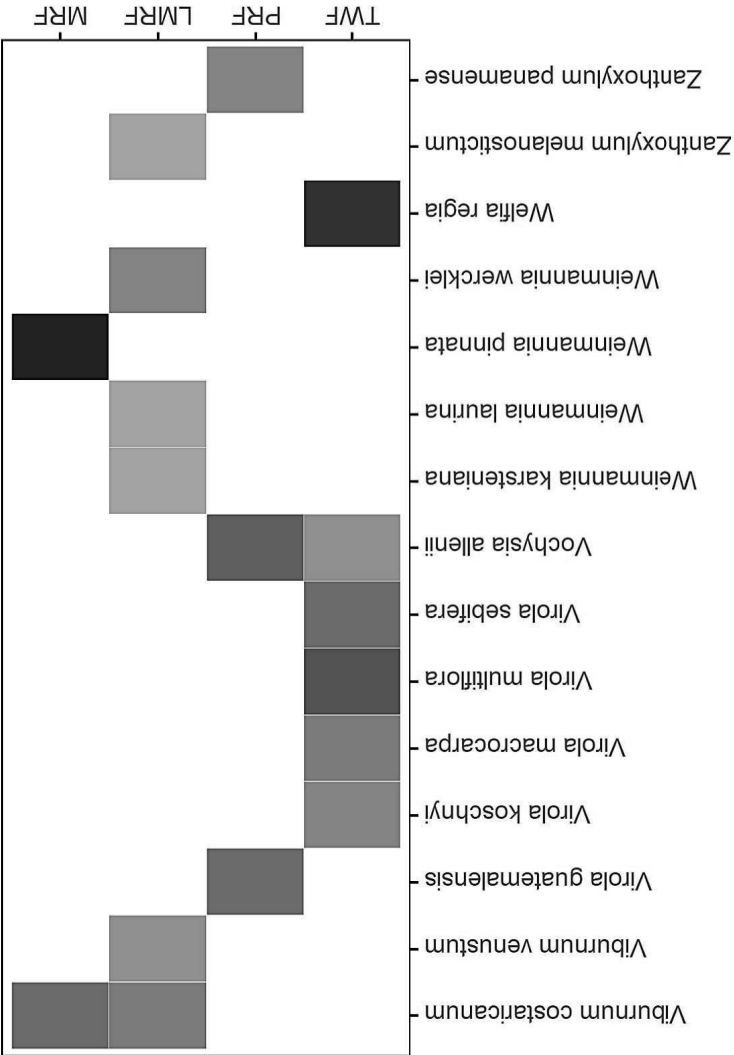

Supplement: Supplementary file 4 [file ECE3-9-5720-s004.pdf]
